# Organ donation after euthanasia in a patient living with dementia: a landmark case report

**DOI:** 10.3389/frdem.2023.1287236

**Published:** 2023-12-05

**Authors:** Nathalie van Dijk, Fabiënne Vliegen, Angelique Ham, Wim de Jongh, Freek Jan Berkhout, Jeroen Blankevoort, Rankie ten Hoopen, Jan Bollen, Walther van Mook

**Affiliations:** ^1^Department of Intensive Care Medicine, Maastricht University Medical Center+, Maastricht, Netherlands; ^2^Faculty of Law, Maastricht University, Maastricht, Netherlands; ^3^Department of Organ Donation Coordination, Amsterdam University Medical Center, Amsterdam, Netherlands; ^4^Department of Organ Donation Coordination, Maastricht University Medical Center+, Maastricht, Netherlands; ^5^Expertisecentrum Euthanasie (EE), The Hague, Netherlands; ^6^Department of Neurology, Flevo Hospital, Almere, Netherlands; ^7^Department of Anesthesiology, Pain and Palliative Medicine, Radboud University Medical Center, Nijmegen, Netherlands; ^8^Department of Intensive Care Medicine, Maastricht University Medical Center+ and School of Health Professions Education, Maastricht University, Maastricht, Netherlands

**Keywords:** euthanasia, dementia, organ donation, advance directive, legislation, case, advance care

## Abstract

**Background:**

Organ donation after euthanasia (ODE) has been performed over 100 times in the Netherlands, primarily involving patients suffering from a neurodegenerative or psychiatric disease. In recent years, the number of euthanasia cases related to dementia has increased in the Netherlands, with some patients living with dementia expressing a wish for organ donation after euthanasia.

**Methods:**

We describe a unique case of a 67-year-old female diagnosed with primary progressive aphasia as part of frontotemporal dementia who requested and underwent organ donation after euthanasia.

**Results:**

The patient had expressed her explicit wishes for both euthanasia and organ donation, which were discussed with her family physician, the Euthanasia Expertise Center (EE), and an organ donation coordinator. The patient was informed that to proceed with ODE, she should still be capable of voicing a voluntary and well-considered request for organ donation. The legally required euthanasia assessment procedure was carefully completed before ODE. Multiple healthcare professionals assessed the patient's competence, voluntariness, and unbearable suffering. Thereafter the patient's ODE request was granted, and both lungs and kidneys were successfully donated and transplanted. *Post hoc* analysis confirmed that all due diligence criteria for euthanasia were met, and the patient's relatives received an anonymous letter of gratitude from one of the organ recipients.

**Conclusions:**

This unique case demonstrates that ODE is feasible from medical, ethical, and legal perspectives in patients living with dementia. This case highlights several aspects essential to enable an ODE request by a patient living with dementia to be granted, such as the role of the physician performing euthanasia, the relevance of the decision-making capacity of the patient, the presence of an advance directive, and the involvement of and support by relatives and caregivers. However, several unresolved ethical issues surrounding ODE in patients with dementia, especially in patients with advanced stages of dementia, warrant further exploration, including the timing of discussing organ donation after the initial euthanasia request.

## Introduction

In Belgium, the Netherlands, Canada and Spain it is possible for patients to donate their organs following euthanasia (Organ donation after euthanasia, ODE) (Downar et al., 2019; Mulder et al., 2022). Patients who choose this combined procedure can donate their kidneys, lungs, pancreas, liver and heart, compliant with a donation after circulatory death (DCD) procedure (Bollen et al., 2016). ODE is mainly performed in patients suffering from a neurodegenerative or psychiatric disease—since malignancy is a contraindication (Bollen et al., 2017). The combined procedure is carefully explained in a national ODE guideline (Bollen et al., 2016).

The 2002 Dutch Euthanasia Act stipulates that euthanasia can only be performed if the patient is suffering hopelessly and unbearably, if their request is voluntary and well-considered, if the patient is adequately informed, and if there are no reasonable alternatives. A second—independent—physician must also give a written opinion on whether these criteria have been fulfilled. Subsequently, the euthanasia procedure has to be performed in accordance with the medical protocol, and promptly communicated with the public prosecutor. Euthanasia is also performed in patients who have dementia.

The number of euthanasia cases due to dementia in the Netherlands increased from five in 2005 to 209 in 2021 (Regional Euthanasia Review Committee, 2021; Groenewoud et al., 2022). Euthanasia was primarily performed in patients diagnosed with dementia who were still in a lucid state and capable of expressing their wishes for euthanasia, with their decision-making capacity being considered intact regarding this matter (see Discussion). In 2021, six of the 209 cases concerned patients with advanced dementia (on a total of 7,666 euthanasia procedures that year) (Regional Euthanasia Review Committee, 2021). Those six patients could no longer communicate their request. In those cases, the advance directive was decisive in establishing whether the request was voluntary and well-considered. In these cases, it has to be clear to the performing physician that the patient is still suffering.

An example of such a patient was the 2016 so-called “coffee euthanasia case” which led to much discussion among healthcare providers (Asscher and van de Vathorst, 2020). In summary, in 2012, a patient diagnosed with severe dementia had written an advance euthanasia directive, which she reconfirmed in 2015 when she was considered still competent by her treating physicians. In January 2016, the patient was considered incompetent regarding her euthanasia request. Two independent physicians confirmed that the patient's euthanasia request met all due care criteria. The patient suffered from agitation, restlessness, stress, fear, sadness, angriness and panic. To avoid confusion and resistance, the euthanizing physician sedated the patient by mixing a sedative in the patient's coffee before administering the euthanasia drugs in 2016 (Asscher and van de Vathorst, 2020). The Regional Review Committees for Termination of Life on Request and Euthanasia, and later the medical disciplinary court, ruled that there were concerns, respectively in 2016 and 2019, which were mainly related to the wording of the advance euthanasia directive. They also addressed the lack of communication with the patient, including oral confirmation of the wish to die and the fact that the euthanasia procedure was performed without the patient being aware of the physician adding a sedative to the patient's coffee. In 2020, the Supreme Court acquitted the doctor, which found that all due care criteria had been fulfilled (Haag, 2019; Asscher and van de Vathorst, 2020; Hoge Raad, 2023). The discourse resulted in the guideline by the Royal Dutch Medical Association on euthanasia in patients living with dementia 2021 (Royal Dutch Medical Association, 2021).

## Should organ donation after euthanasia also be possible in patients who have dementia?

From the perspective of this legal process, and given the contested ethical status of euthanasia for patients who have dementia, adding organ donation may further complicate the moral landscape. How should healthcare professionals respond to patients suffering from less or more advanced dementia, requesting organ donation after euthanasia, with or without having written an advance directive? Although ODE in dementia patients has already been performed in the Netherlands, cases of organ donation after euthanasia in dementia patients have not been reported in the scientific literature.

This article, supported by the patient's relatives, provides insights into how a dementia patient's last wish for ODE can be granted within the legal and ethical boundaries, scaffolded around an illustrative practice case. The CARE case report guidelines were used to assure the accuracy, transparency, and usefulness of the case herein described (CARE Guidelines, 2023).

## Case report

In 2018, a 67-year-old female former healthcare professional was diagnosed with primary progressive aphasia, a subtype logopenic variant, as part of frontotemporal dementia after an extensive workup following an emergency room visit to a neurologist for sudden confusion. Her prior medical history was unremarkable. Her mother, sister and aunts were diagnosed with dementia. Having experience with the poor prognosis and possible rapid decline in quality of life both as a former nurse and informal caregiver to her mother and sister, she readily consulted her general practitioner. She discussed her explicit wish for euthanasia in due time, which she also documented in writing to inform her friends and relatives.

The family physician subsequently consulted the EE. This specialized center counsels and supports physicians who are helping patients with a euthanasia request and gives care to patients who have a euthanasia request. The patient also voiced her explicit wish to donate her organs during this initial meeting with the physician of EE and therefore, requested the euthanasia procedure to be performed in the hospital. Her wishes were quoted: “*adequately functioning organs should not simply be discarded*” and “*others without adequately functioning organs should be given a chance to live on somewhat longer*.” She had registered as a potential organ donor decades before. The consulted organ donation coordinator extensively explained the ODE procedure to the patient and her relatives, after exploring the patient's medical history and social context, and found the patient and her relatives motivated and supportive of a possible ODE procedure. The organ donation coordinator confirmed the patient's registration in the national donor registry. The patient and her husband were informed that for ODE to be performed, the patient should still be able to voice a voluntary and well-considered request for organ donation, so timing such a request *before* becoming incapacitated was considered necessary.

It was also explained that the legally mandated euthanasia procedure should be completed first. The EE physician was not present during this explanatory meeting, to clearly separate the discussions on euthanasia and organ donation. After that, the EE's consulting physician and nurse visited the patient regularly, ten times, in a 2½ year period. Then, after the 10th house call, the patient voiced that the euthanasia wish had become urgent and topical. Over these years, she had both physically and mentally deteriorated, with increasing aphasia, inability to perform daily tasks of living, and increasing dependency on informal caregivers, including her husband. Dressing, cooking, gardening, making electronic payments, and driving a car had become impossible, and holidays had become too confusing. She described her situation as miserable and was heartbroken. During the dialogues, the patient's responses to questions remained always adequate and to the point, and there was never any doubt regarding the euthanasia request. Her expression and state of mind were always congruent with the topic discussed. When discussing the euthanasia request, she voiced that she had cared for dementia patients and considered the prospect of further deterioration unacceptable. She stated “*I do not want to go to such a nursing home, never ever!*” And the answer to the question of what time frame she had in mind regarding the euthanasia procedure: “*Soon!*”, reasoning from the same perspective. She was fully aware of the consequences of her euthanasia request, if granted, namely death. Her husband respected her wishes.

The consultation of an external physician, as legally mandated, confirmed that the euthanasia request had been made voluntarily, and well considered. The dementia was progressive and untreatable, so the situation was hopeless, but also unbearable considering the prospect of further deterioration, the inability to continue to perform the things she loved, and the prospect of future admission to a nursing home. All due diligence criteria regarding the euthanasia request were thus fulfilled.

Thereafter, the preparatory investigations required for a donation procedure were performed during several hospital visits and two house calls. Considering her hindering aphasia, the patient proactively informed her friends and loved ones in a letter about her progressive debilitating illness, her reasons for requesting euthanasia, and the date set for the euthanasia procedure. She herein also explained her wish for organ donation and the practical implications, namely admission to the hospital. During the subsequent ODE procedure, both lungs and kidneys were donated and transplanted. All donated organs were transplanted with good graft function. The *post hoc* Regional Review Committee confirmed that all due diligence criteria had been met and that the euthanasia procedure had been performed compliant with the Dutch euthanasia act. The relatives received an anonymous letter of gratitude from one of the recipients.

## Discussion

This illustrative case, although not the first case of organ donation after euthanasia in a patient with dementia, is the first case published and with full support by the relatives. In the paragraphs below we subsequently discuss aspects of the process relating to the euthanasia procedure, respectively the organ donation request, representing the actual historical timeline of events.

### Role of the euthanizing physician

In the case herein described, the family physician referred to the EE, where the euthanizing physician was employed. In a review of 111 published Dutch euthanasia due to dementia case summaries, most patients discussed their first euthanasia intention with their family physician, who in 39.6% declined assistance (Groenewoud et al., 2022). In 40% of these 111 performed euthanasia cases, the euthanizing physician was a physician from the EE (Groenewoud et al., 2022). The review however also mentions that the euthanizing physician had “no prior patient-doctor relationship.” The euthanizing physician in our case had performed a long-term 2 ½ year medical follow-up of the patient's request for euthanasia and the longer follow-up period with 10 contacts, including multiple house calls, facilitated the euthanizing physician regarding an adequate assessment of the due diligence criteria for euthanasia, and the timing of the actual euthanasia procedure when all due diligence criteria were fulfilled. As mandated by law, consultation of a qualified external physician ensured that objectivity is maintained regarding the assessment of the ultimate euthanasia request.

### Competence and voluntariness of the patient's request

The assessment of decision-making capacity in dementia patients regarding euthanasia, organ donation and the combination is a complex and sensitive process. Decision-making capacity patients can fluctuate in patients living with dementia, and thus the assessment is time sensitive, and may vary according to the stage of dementia and the type of decision (Trachsel et al., 2015). The assessment process should be conducted while respecting the patient's autonomy, dignity, and consideration of their best interests. The process involves several sequential steps, including establishing an individual's baseline cognitive and functional abilities, including speaking with caregivers and family members, providing information in a way the patient can understand, and subsequent assessment of their understanding. In addition, it should be carefully assessed whether the patient can appreciate the consequences of their decision, and communicate their decision clearly and coherently. The decision should thereafter be carefully documented. In the case herein described, the general steps were all carefully followed, and documented as such in the patient's case file.

In the above-mentioned review of 111 publicly available case summaries of euthanasia in patients living with dementia, 87 of the 111 patients were deemed competent enough to make an end-of-life decision (Groenewoud et al., 2022). Twelve (10.8%) were found to have been not fully competent and the decision for euthanasia was found to be based on fragmented expressions, circumstantial evidence and written and/or oral directives. In addition, in 10 out of these 111 cases, the information about the nature and severity of suffering, and the request came at least partly, or in full, from the relatives (Groenewoud et al., 2022).

The other approach was to act primarily on an advance directive, often interpreted by the relatives. In our case the patient was both competent at the initial request, in the years after the initial request, and when ultimately requesting the euthanasia to be performed. A formal advance directive was not available, yet the patient, assisted by her husband, chose to write down her explicit request for euthanasia and wish for the subsequent organ donation to inform friends and relatives before the euthanasia procedure, considering her inability to do so verbally due to the progressive aphasia.

### Unbearability of suffering

Our patient was suffering unbearably from both her physical and mental deterioration due to progressive dementia, but also from the prospect of further deterioration, not merely from the absence of any prospect for improvement. Groenewoud et al. (2022) recently stated that more than in terminal illness (e.g., due to malignancies), the suffering in dementia differs per patient, depending on the patient's personality, biography and background (Groenewoud et al., 2022). In our patient, her clearly articulated early wish for euthanasia was at least partly based on her experiences with dementia in other people close to her. Such “ghosts from the past” made such a deep impression that “the prospect of ending up in a similar situation causes unbearable suffering in the present” (Groenewoud et al., 2022). Many patients fear losing control, ceasing to be the person they are, losing their dignity, and/or the prospect of having to move to a nursing home.

Groenewoud et al. described two preventive strategies in this regard: writing an advance directive with a list of the conditions one wants to forego, respectively to undergo euthanasia at an earlier stage. Whereas in their study, in 70% of case summaries studied, patients had “*licensed*” their physicians and/or relatives to make a euthanasia decision on their behalf in case of mental incompetence, patients also realized that such so-called “*now for then*” preferences will not hold under all circumstances (Groenewoud et al., 2022). Although our patient had not drafted a formal advance directive (“only” an explanatory letter to relatives and friends), the 2½ year process facilitated determining the patient “*optimal*” timing for euthanasia and organ donation: not too early from the perspective of absence of unbearability of suffering and quality of life, and not too late from the perspective of being incapacitated or incompetent to repeat their earlier request due to as some argue “lack of continuity of personality” (de Boer et al., 2010; Shaw, 2012). It should, however, be acknowledged that in the presence of an advance directive in a patient with advanced dementia, yet in the absence of unbearable and hopeless suffering, a euthanasia request will not be granted, since the due diligence criteria for euthanasia are not met (Hoge Raad, 2023).

### The relatives and caregivers

The endorsement of the ODE procedure by the caregivers and relatives was not under pressure in the case herein described. In the 2022 review of 111 published euthanasia in dementia cases, this was, however, the case in 12.6% of cases, either directly and orally or indirectly through aggressive behavior (Groenewoud et al., 2022). In 5.4% of cases, the patients were reported to consider suicide if their death wish was not granted (Groenewoud et al., 2022), perhaps also directly or indirectly pressuring the relatives. In the case herein presented, the relatives fully supported the patient's wish and request for euthanasia, as well as organ donation. Formally, however, legal permission of the relatives for the ODE procedure is *not* required.

### The request for organ donation after euthanasia

A euthanasia screening procedure and a dialogue regarding organ donation usually are strictly independent and separated. Even more strict, the Dutch ODE guideline mentions that the patient's request for organ donation should be made independently and may not be raised by the treating physician (Nederlandse Transplantatie Stichting, 2023). The latter potentially conflicts with the principles of providing adequate patient information and shared decision-making. However, in our experience, many patients requesting euthanasia, also mention organ donation *early* in the contacts with their treating physician. This was also the case in our patient. At the patient's request, all information about an ODE procedure was provided to the patient and her relatives during an early house visit by the organ donation coordinator. Subsequently, the national donor registry was consulted. At the time the euthanasia request of our patient was made, the Royal Dutch Medical Association guideline on euthanasia in patients with (advanced) dementia (Royal Dutch Medical Association, 2021), based on the 2020 Supreme Court ruling (Hoge Raad, 2023), was not yet available in the Netherlands. Consequently, at the time, in 2019, the donation coordinator reported that once a stage of advanced dementia had been reached, ODE would no longer be an option. In contemporary practice, a euthanasia request based on an advance directive by a patient with advanced dementia suffering unbearably and hopelessly could be granted, if the due care criteria for euthanasia are met.

### Lessons learned on ODE in dementia

Several lessons can be learned from the case summary in this article.

Patients living with dementia may suffer unbearably from the actual physical and mental deterioration due to progressive dementia, but also from the prospect of future deterioration, not merely from the absence of any prospect for improvement. A clearly articulated wish for euthanasia in dementia is often based on prior experiences with dementia in people close to them. Early contact of the patient and relatives with a physician with expertise in euthanasia and longitudinal follow-up of the patient's condition and request ensures an early open ear and continuity of support and uniformity of contact person thereafter.

To facilitate adequate shared decision-making, an organ donation coordinator can provide the patient and relatives early with information on the organ donation procedure after euthanasia, if requested by the patient. The premise is that the patient's preferences as registered in the national donor registry match the patient's verbal request for organ donation.

An advance directive is not absolutely necessary in case of an early euthanasia request in a patient living with an early stage of dementia. However, an advance directive may become practically required, indispensable, and decisive once a dementia patient becomes incapacitated in the course of illness, and the competence, voluntariness and unbearability of suffering may be questioned. And perhaps most importantly, the patient's continuous support by the patient's loved ones and relatives is paramount during all phases of the euthanasia request process. However, formal legal permission of the relatives for the ODE procedure is not required.

## Strengths and limitations

This publication is the first to touch upon organ donation after euthanasia in a patient living with dementia in general, and in the Netherlands specifically. This landmark publication may therefore be of interest and relevance for patients, their relatives and healthcare professionals caring for patients living with dementia. Its primary purpose is to raise awareness regarding the so far relatively unknown option of organ donation in this patient category, thereby facilitating future scientific and societal debate on this topic. Considering the preliminary nature of this publication, the topic of ODE in dementia necessitates further in-depth medical, ethical and legal exploration, as previously touched upon by Groenewoud et al. (2022). Remaining issues include, for example: What are the attitudes of health care professionals involved in elderly care toward organ donation in patients with dementia? What are their experiences and perceived needs regarding ODE? Who can introduce organ donation as a topic of discussion: patients, relatives, and/or treating physicians? And when: should this be done after the euthanasia request is granted, or is an earlier dialogue from the perspective of advance care planning and shared decision making also acceptable? Addressing these and other topics, although of paramount importance, is beyond this article's scope.

## Conclusions

To the best of our knowledge, this is the first publication concerning a case of organ donation after euthanasia in a dementia patient in the Netherlands. This combined procedure is feasible from a medical, ethical and legal perspective in this group of patients. Since several important issues continue to be debated, especially in patients with advanced dementia, future larger, in-depth studies are warranted to explore these topics.

## Data availability statement

The raw data supporting the conclusions of this article will be made available by the authors, without undue reservation.

## Ethics statement

Written informed consent was obtained from the individual(s) for the publication of any potentially identifiable images or data included in this article.

## Author contributions

AH: Resources, Writing – review & editing. FB: Data curation, Writing – review & editing. FV: Data curation, Writing – review & editing. JBo: Conceptualization, Data curation, Writing – original draft. JBl: Data curation, Writing – review & editing. ND: Conceptualization, Writing – original draft, Writing – review & editing. RH: Writing – review & editing. WJ: Conceptualization, Data curation, Writing – original draft. WM: Conceptualization, Supervision, Writing – original draft.
